# Role of BNST CRFR1 Receptors in Incubation of Fentanyl Seeking

**DOI:** 10.3389/fnbeh.2020.00153

**Published:** 2020-08-28

**Authors:** Utsav Gyawali, David A. Martin, Agnieszka Sulima, Kenner C. Rice, Donna J. Calu

**Affiliations:** ^1^Program in Neuroscience, School of Medicine, University of Maryland, Baltimore, Baltimore, MD, United States; ^2^Department of Anatomy and Neurobiology, School of Medicine, University of Maryland, Baltimore, Baltimore, MD, United States; ^3^Intramural Research Program, National Institute on Drug Abuse, National Institute on Alcohol Abuse and Alcoholism, Baltimore, MD, United States

**Keywords:** incubation, opioid dependence, withdrawal, CRF, BNST

## Abstract

The time-dependent increase in cue-triggered opioid seeking, termed “incubation of opioid craving,” is modeled in rodents by examining responding for opioid-associated cues after a period of forced abstinence. With opioid drugs, withdrawal symptoms may heighten cue reactivity by recruiting brain systems involved in both reward seeking and stress responses. Corticotropin releasing factor (CRF) in the bed nucleus of the stria terminalis (BNST) is a critical driver of stress-induced relapse to drug seeking. Here, we sought to determine whether BNST CRF receptor 1 (CRFR1) signaling drives incubation of opioid craving in opioid dependent and non-dependent rats. First, we tested whether BNST CRFR1 signaling drives incubation of opioid craving in rats with short-access fentanyl self-administration experience (2.5 μg/kg/infusion, 3 h/day for 10 days). On Day 1 of forced abstinence, we gave bilateral intra-BNST vehicle injections to all rats and measured lever responding for opioid cues in the absence of fentanyl infusions. On Day 30 of forced abstinence, we gave an identical test after bilateral intra-BNST injections of vehicle or CRFR1 receptor antagonist, R121919 (1 μg/0.3 μL/hemisphere). Vehicle treated rats showed greater responding for opioid associated cues on Day 30 relative to Day 1, and this incubation effect was prevented by intra-BNST R121919 on Day 30. Next, we incorporated an opioid-dependence procedure to investigate whether BNST CRFR1 signaling drives opioid cue-reactivity to a greater extent in opioid-dependent relative to non-dependent rats. We trained rats to self-administer fentanyl for 5 days before initiating the dependence phase and resuming daily fentanyl self-administration sessions for 10 days. We gave intra-BNST R121919 or vehicle injections before testing during acute (Day 5) or protracted (Day 30) withdrawal. During acute withdrawal, antagonizing BNST CRFR1 decreased the number of press bouts without affecting bout size or duration. These patterns of responding with R121919 treatment resulted in less fentanyl-associated conditioned reinforcement during test. Together, these findings suggest a role for BNST CRFR1 signaling in driving cue-reinforced opioid seeking after periods of forced abstinence.

## Introduction

Substance use disorder (SUD) consists of cycles of compulsive drug seeking and consumption, followed by periods of abstinence, withdrawal, and relapse (Koob and Volkow, 2016). Drug-associated cues trigger intense craving for drugs of abuse even after periods of abstinence (Sinha, 2011; Li et al., 2015). With opioid drugs of abuse, abstinent opioid-dependent individuals also experience aversive withdrawal symptoms that promote relapse (DSM-V). Preclinically, the incubation of drug craving model examines cue-triggered drug seeking in rodents after a period of forced abstinence (Grimm et al., 2001; Pickens et al., 2011; Reiner et al., 2019). During abstinence from opioid drugs of abuse, withdrawal may invigorate cue-driven opioid seeking by recruiting brain systems involved in both stress responses and reward seeking. The bed nucleus of the stria terminalis (BNST) is a critical regulator of both stress states and reward related behavior (Ch’ng et al., 2018). Corticotropin releasing factor (CRF) in the BNST is elevated during acute ethanol withdrawal (Olive et al., 2002) and CRF mRNA is increased in the BNST by stress-induced reinstatement of heroin seeking after periods of forced abstinence (Shalev et al., 2001). We postulate that CRF system in the BNST regulates incubation of opioid craving after forced abstinence in opioid dependent and non-dependent rats.

The CRF system is heavily implicated in opioid dependence and withdrawal (Sarnyai et al., 2001; Logrip et al., 2011). CRF receptor 1 (CRFR1) is necessary for the expression of conditioned behaviors associated with opioid withdrawal states (Contarino and Papaleo, 2005). Systemic CRFR1 antagonism reduces behavioral signs of precipitated opioid withdrawal (Iredale et al., 2000). In addition, CRFR1 antagonist dose dependently reduces heroin intake only in long access rats that are postulated to be heroin dependent (Greenwell et al., 2009). Together, these studies suggest that CRFR1 antagonists could be reducing heroin seeking by alleviating a negative emotional state in heroin dependent rats. When interpreting these CRF system findings with regard to relapse studies, it is critically important to consider whether withdrawal was precipitated or spontaneous. While precipitated opioid withdrawal is insufficient to reinstate heroin seeking, both stress and heroin priming reinstate opioid seeking after spontaneous withdrawal (Shaham and Stewart, 1995).

Several studies point to BNST CRF in stress-induced reinstatement of drug seeking, and specifically, a role of BNST CRFR1 signaling (Erb et al., 2001; Mantsch et al., 2016). Early studies showed intracerebroventricular pretreatment of a CRFR1 antagonist prevents stress-induced reinstatement of morphine conditioned place preference (Lu et al., 2000) and this effect is mediated by BNST (Wang et al., 2006). Central amygdala (CeA) CRF projections to the BNST mediate stress-induced reinstatement of cocaine seeking (Sakanaka et al., 1986; Erb et al., 2001). Stress-induced reinstatement only occurs in the presence of cues associated with drug self-administration suggesting an interplay between stress and drug-associated cues (Shelton and Beardsley, 2005). Incubation of opioid craving studies have yet to address whether spontaneous opioid withdrawal potentiates the motivational properties of drug-associated cues. In the present study, we begin to address this by examining cue-reinforced opioid seeking after forced abstinence in opioid-dependent and non-dependent rats. Further, we seek to identify whether BNST CRFR1 receptors are involved in incubation of opioid craving.

Here, we sought to determine whether BNST CRFR1 signaling drives incubation of opioid craving in opioid-dependent and non-dependent rats. First, using CRFR1 antagonist, R121919 [(2,5-dimethyl-3-(6-dimethyl-4-methylpyridin-3-yl)-7 dipropylamino pyrazolo[1,5-a]pyrimidine] (Heinrichs et al., 2002; Jagoda et al., 2003), we tested whether BNST CRFR1 signaling drives incubation of opioid craving in rats with short-access fentanyl self-administration experience. Second, we incorporated an opioid-dependence procedure to investigate if BNST CRFR1 mediates opioid cue-reactivity to a greater extent in opioid-dependent rats compared to non-dependent rats. Finally, to determine whether intra-BNST R121919 affected opioid consumption or the ability to lever press we utilized within-session behavioral economic opioid demand procedures. These experiments allow us to test the role of BNST CRFR1 in mediating incubation of fentanyl seeking after acute and protracted withdrawal in opioid-dependent and non-dependent rats.

## Materials and Methods

*Subjects*: We used 8 weeks old male Sprague Dawley rats (Charles River, *n* = 124) weighing 250–350 g before surgery and maintained the rats under a reverse 12:12 h light-dark cycle (lights on and off at 9 AM and 9 PM, respectively) with food and water available *ad libitum*. We performed the experiments in accordance to the “Guide for the care and use of laboratory animals” (8th edition, 2011, US National Research Council) and experimental protocols were approved by the University of Maryland Institutional Animal Care and Use Committee. We excluded rats because of failure of catheter patency (*n* = 48) or incorrect cannula placements (*n* = 4).

### Surgery

We performed a single surgery to implant both intravenous catheters and intracranial cannulae.

*Catheterization surgery*: We anesthetized 9 week old rats with isoflurane (4.5% induction, 2-3% maintenance) and implanted catheters into the right jugular vein as described in Martin et al. (2020). Following surgery, we injected rats daily with 0.05 mL of anti-microbial, anti-bacterial, and anti-coagulant Taurolidine-Citrate (TCS) catheter lock solution i.v. (Cat# TCS-04, Access Technologies, IL, United States) to reduce biofilm and clot formation, to promote catheter patency, and to reduce the risk of microbial infection. We checked catheter patency occasionally by giving intravenous injections of 0.1 mL of methohexital sodium. Rats without a sudden loss of muscle tone were removed from the study.

*Intracranial surgery*: We implanted bilateral guide cannulae (23 gage; Plastics One) 1.0 mm above the BNST. We implanted cannulae at AP: −0.3 mm, ML: ±3.6 mm, DV: −6.2 mm (18° angle) from Bregma and anchored the cannula to the skull using dental cement and jeweler’s screws. We used the above coordinates based on a previous study (Pomrenze et al., 2019) and pilot experiments. After 1 week of recovery from surgery we started the self-administration training phase.

*Intracranial and subcutaneous injections*: We intracranially injected 0.3 μL/side of the selective CRF1 receptor antagonist R121919 [3-(6-(dimethylamino)-4-methyl-pyrid-3-yl)-2,5-dimethyl-*N*,*N*-dipropyl-pyrazolo(2,3-a)pyrimidin-7-amine], NBI 30775, a gift from Dr. Kenner Rice (Chemical Biology Research Branch, Drug Design and Synthesis Section, National Institute on Drug Abuse and National Institute on Alcohol Abuse and Alcoholism, Rockville, MD, United States) into the BNST 15 min before the beginning of the test session. We dissolved R121919 in 4% Kolliphor RH40 in aCSF, pH 5 (Pomrenze et al., 2019). We injected 4% Kolliphor RH40 in aCSF as vehicle (Day 1, Day 5, or Day 30 tests) or R121919 (Day 5 or Day 30 tests). We performed all intracranial injections over 1 min with injectors that extended 1 mm below the tips of guide cannulae. We left the intracranial injectors in place for an additional minute to allow for diffusion.

*Apparatus*: We trained rats in self-administration chambers housed in sound attenuating cabinets (Med Associates) containing two retractable levers (Active and Inactive) on the same wall located 10 cm above the chamber floor. We counterbalanced the position of the Active and Inactive Levers across rats.

*Drugs*: We purchased fentanyl citrate from Sigma Aldrich or Cayman Chemical and diluted it in 0.9% sterile saline at 12.5 or 1 mg/mL. We purchased remifentanil from Toronto Research Chemicals, morphine sulfate from Spectrum Chemical, and methohexital sodium from Parchem. We diluted remifentanil and morphine in 0.9% sterile saline at 12.5 and 50 mg/mL, respectively.

*Self-administration training*: We trained rats in daily sessions to self-administer fentanyl (for dose see Drugs section for detail) for 3 h per day under an FR1-20s timeout reinforcement schedule. Presses on the Active Lever resulted in activation of a syringe pump which delivered a 28 μL infusion of fentanyl over one second paired with a compound 5-s light (7.5 W white light located above the Active Lever) and a tone (2,900 Hz speaker located above the light) cue. A red houselight remained on during the entire session. Presses on the Inactive Lever were recorded but not reinforced.

*Day 1, 5 or 30 extinction testing*: We started the 90-min extinction tests 15 min after intracranial injections. For experiment 1, rats (*n* = 28) completed the extinction test on Day 1 and Day 30 while for experiment 2, rats completed the extinction test either on Day 5 only (*n* = 28) or Day 5 and Day 30 (*n* = 16). All extinction sessions began with extension of Active and Inactive Levers and illumination of red house light which remained on for the duration of the session. Presses on the Active Lever no longer resulted in drug infusions, but still resulted in contingent tone-light cue on the same FR1 20 s time out schedule of reinforcement used during training. Maintaining the same reinforcement schedule during testing enables us to examine patterns of drug seeking based on previously learned cue reinforcement contingencies in the absence of the drug, instead of newly acquired cue reinforcement contingencies in the absence of the drug. We recorded number of active presses for all experiments as well as the timestamp of each active press during day 5 test. Presses on the Inactive Lever were recorded but had no consequences.

*Histology*: After testing, we deeply anesthetized rats with isoflurane and transcardially perfused them with 100 mL of 0.1 M PBS followed by 400 mL of 4% paraformaldehyde (PFA) in dH_2_O. We extracted the brains and post fixed them in 4% PFA for 2 h before we transferred them to 30% sucrose in PBS for at least 48 h at 4°C. We subsequently froze the brains and stored them in −20°C until sectioning. We sectioned the brains at 40 μm containing BNST on a cryostat (Leica Microsystems) and collected every third section through the cannula placement in a cryopreservant. Finally, we stained the sections with cresyl violet and coverslipped with Permount. We verified cannulae placements under a light microscope.

*Statistical analysis*: We organized the data in Excel and analyzed it using SPSS, Prism, and Matlab. We used mixed design repeated measures ANOVAs to analyze the self-administration training data, separately examining number of cues + infusions earned and lever press (Active and Inactive) data, using within- and/or between- subject factors of Session and Dependence Group as appropriate. For Experiment 1 incubation data, we separately examined cues earned and lever press data in mixed design repeated measures ANOVA using Withdrawal Day (Day 1, Day 30), Lever (Active, Inactive) and Day 30 Treatment (vehicle, R121919) as within- or between-subject factors as appropriate. For Experiment 2, Day 5 vs Day 30 protracted withdrawal test data we performed similar analyses on cues earned and lever data adding Dependence (dependent, non-dependent) as a factor. For Experiment 2, Day 5 acute withdrawal test data, we examined cues earned and lever data using Day 5 Treatment and Dependence with Lever added as appropriate. Further, for the day 5 test we collected active press timestamp data to conduct a press bout analysis. We defined a press bout as two or more presses for which the interval between successive presses in the bout did not exceed the time out interval of 20 s. We calculated number of bouts, presses per bout, bout duration (time from first to last press in a bout) and inter bout interval (time between last press in bout and first press in next bout).

*Demand data analysis*: We analyzed demand data as previously described (Martin et al., 2020). In brief, we used drug consumption for each bin as the primary dependent measure. We defined the price of the drug as the number of responses required to reach 20 μg/kg such that price units = number of responses/20 μg/kg. Unlike our previous study, we also included data from the first bin as we did not observe a “loading effect” with remifentanil. We averaged the consumption and price across adjacent 15-min bins, 1–2, 3–4, 5–6, 7–8, 9–10 resulting in five prices and five consumption values for each rat. We fitted the data in Matlab using the “fitnlm” function modeled with an exponential demand equation *Q* = *Q*_0_×10^k(e−αQ_0_C_−1_)^. *Q*_0_ represents the theoretical consumption of drug at low prices when no effort is required, *α* is the measure of demand elasticity and is inversely related to motivation, *Q* is the consumption at a given *C* (price) during a particular bin, and k is the logarithmic range of consumption data. We constrained *Q*_0_ values to three times the maximum consumption to reduce overestimation of *Q*_0_ in sessions where responding falls quickly with increasing price. Constraining *Q*_0_ resulted in insignificant decrease in *R*^2^ values (average *R*^2^ for constrained: 0.948 and unconstrained: 0.950). We used a *k* value derived from each session’s consumption data as described in Martin et al. (2020) to maximize the quality of fit and to avoid systematic errors in *α*. We excluded three sessions due to low *R*^2^ values (*R*^2^ < 0.25) while calculating *Q*_0_ and *α* from demand graphs. We compared *Q*_0_, *α*, total Active Lever presses, and total consumption between days rats received intra-BNST vehicle or intra-BNST R121919. We performed paired *t* test to determine if above parameters were significantly different in drug injected day compared to vehicle injected day.

#### Experiment 1

After a week of recovery following surgery, we trained rats to self-administer fentanyl for 10 days as described above. For the first cohort of animals (*n* = 12) we trained rats to self-administer fentanyl (Sigma Aldrich) at 2.5 μg/kg/infusion. For the second cohort (*n* = 16), fentanyl was not available from our original source. Fentanyl sourced from Cayman Chemical visibly occluded rats from responding at 2.5 μg/kg/infusion. Thus, for cohort 2 we reduced the concentration of fentanyl (Cayman Chemical) to 2.0 μg/kg/infusion for days 3 and 4 of self-administration and to 1.5 μg/kg/infusion for the rest of the self-administration phase. Since the terminal levels of responding for both cohorts were not significantly different (see section “Supplementary Results”), we pooled the data together. During training, Active Lever presses resulted in fentanyl infusion paired with a compound 5s tone-light cue located above the Active Lever.

*Incubation extinction tests*: After 10 days of training, we injected vehicle intracranially into the BNST of all rats 15 minutes prior to Day 1 of forced abstinence extinction test. After 30 days of forced abstinence (Day 30), we retested the same rats and injected either intra-BNST vehicle in *n* = 13 rats or intra-BNST R121919 in *n* = 15 rats in a mixed within-between subject design.

#### Experiment 2

After a week of recovery following surgery, we trained rats to self-administer fentanyl similar to *Experiment 1* for 5 days before dependence induction. Similar to *Experiment 1*, *n* = 14 animals were trained in 1.5 μg/kg/infusion. The terminal levels for responding were not significantly different in this cohort as well (see section “Supplementary Results”) so we pooled the data together.

*Dependence phase and ongoing self-administration*: After 5 days of self-administration, we gave rats twice daily injections (morning and evening; every 12 ± 2 h) of either saline (non-dependent) or ascending doses of morphine (dependent). The morphine dosing regimen was 10, 20, 30, 40, 50, 60, and 70 mg/kg subcutaneous injections for 7 days (Cooper et al., 2008). We recorded rats’ baseline weight at the end of 5 days of self-administration and also recorded their weights before morphine injections twice daily. We averaged the twice daily weights (Days 1–7) and also recorded weights once daily during self-administration post-dependence (Days 8–17). We calculated change in body weight by subtracting their daily weights from their baseline weight. On the 7^th^ day of dependence phase, we injected the dependent rats with 70 mg/kg morphine only in the morning and saline in the evening before resumption of daily fentanyl self-administration training the next day. We trained rats to self-administer fentanyl for 10 more days while they were maintained on once daily saline or morphine (40 mg/kg s.c) injection after the end of each self-administration session. In an attempt to reduce the confound of differences in fentanyl consumption and in cue exposure for dependent and non-dependent rats, we established infusion maximum (*I*_max_), the maximum number of infusions per session, during the post-dependence ongoing self-administration sessions. Once the *I*_max_ was met, the program ended, and rats were immediately removed from the self-administration chambers. The infusion maximum for the post-dependence self-administration sessions were *I*_max_ 35 for self-administration sessions 6 and 7, *I*_max_ 45 for sessions 8 and 9, *I*_max_ 55 for sessions 10 and 11, *I*_max_ 65 for sessions 12 and 13, and *I*_max_ 75 for sessions 14 and 15. The last post-dependence self-administration session coincided with last homecage morphine or saline injection. For data analysis, we assigned a value of 180 (the total length of the session) as the latency to reach *I*_max_ for rats that did not reach the *I*_max_.

*Dependence measures*: 24 h after session 15 of post-dependence self-administration, we video recorded rats for 20 min in an operant chamber devoid of levers and cues. We video scored the number of wet-dog shakes; a single wet-dog shake is defined as a rapid bout of alternating head and body shaking lasting less than 2 s, which is a reliable measure of opioid withdrawal symptom (Lee et al., 1989). Two experimenters were blind to the dependence assignment and independently scored a subset (32%) of the same videos to ensure consistency and accuracy. Each individual’s video scoring was significantly correlated (*r*^2^ = 0.92, *p* < 0.001).

*Acute and Protracted extinction tests*: Because the dependence procedures we used result in acute withdrawal symptoms (Cooper et al., 2008), we performed the acute test 5 days after the last post-dependence self-administration session/last homecage morphine or saline injection. For the *Acute Withdrawal (Day 5 test)*, we gave one subset of rats intra-BNST vehicle injections (*n* = 14) or intra-BNST R121919 injections (*n* = 14) prior to extinction test as described above. We compared their extinction responding using a between-subject design. A subset of these rats was then tested in remifentanil demand threshold procedure outlined below. For the *Protracted withdrawal (Day 5 vs Day 30 test)*, we gave a different subset of rats (*n* = 16) intra-BNST vehicle injections on Day 5 and retested half with intra-BNST vehicle injections (*n* = 8) and half with intra-BNST R121919 injections (*n* = 8) prior to extinction test as described above.

*Demand thresholding training and testing*: Following their Day 5 extinction test, we trained the rats (*n* = 12) in the acute withdrawal group from *Experiment 2* to self-administer a short-acting μ-opioid receptor agonist, remifentanil, in a within-session demand thresholding procedure. The drug infusion was paired with the light/tone compound cue on an FR1 schedule. Across the demand thresholding, the duration of each remifentanil infusion and cue was decreased every 15 min on a quarter log scale. Each session tested 10 doses of remifentanil (20, 11.2, 6.4, 3.6, 2, 1.1, 0.64, 0.36, 0.2, 0.1 μg/kg/infusion). The demand thresholding procedure lasted 150 min. A red house light was on for the entire duration of the session, except during the length of an infusion. To establish a baseline, we trained rats in six daily demand thresholding sessions before starting the test sessions. After baseline sessions, we gave within-subject subcutaneous injections of R121919 (10 mg/kg, 2 mL/kg/infusion) or vehicle 1 h before two counterbalanced demand thresholding test sessions (data not shown). Following subcutaneous injection, we injected (*n* = 10 rats) R121919 or vehicle intra-BNST, using the same dose and volume as described previously, 15 min before two counterbalanced demand thresholding sessions.

## Results

### Experiment 1: Incubation of Fentanyl Craving

**FIGURE 1:**
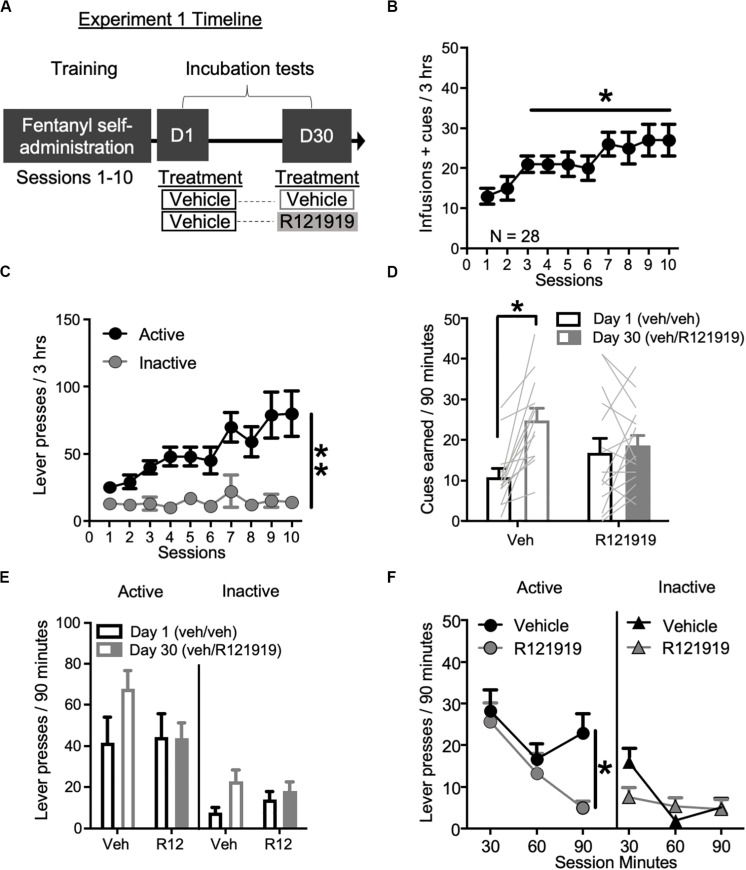
Experiment 1: BNST CRFR1 antagonist effect on incubation of fentanyl craving. **(A)** Experimental timeline: we trained rats to self-administer fentanyl for 3 h/day for 10 days. On forced abstinence Day 1 and Day 30, we measured lever responding under extinction conditions. **(B)** Fentanyl infusions + cues earned across the 10 daily self-administration sessions. **(C)** Rats discriminated Active from Inactive Lever during self-administration training. **(D,E)** Incubation test data showing cues earned on FR1, 20 s TO schedule **(D)** and Active/Inactive Lever pressing **(E)** on Day 1 and Day 30 of forced abstinence for vehicle (open bars) treated and R121919 treated (filled bars) conditions. **(F)** Time course of Active and Inactive Lever presses on Day 30 test for both treatment groups. *p < 0.05, **p < 0.01, different from Session 1 in **(B)**, Lever main effect in **(C)**, different from Day 1 in **(D)**, and Treatment main effect in **(F)**. R12 = R121919. *n* = 13 Veh, *n* = 15 R12. Data are mean ± SEM.

*Self-administration training*: We trained rats 3 h/day for 10 days in fentanyl self-administration. Experimental timeline is shown in Figure 1A and infusions + cues earned data in Figure 1B. We observed main effect of Session (1–10) on infusions + cues earned (*F*_9_,_243_ = 6.83, *p* < 0.001), demonstrating rats consumed more fentanyl and were exposed to more cue pairings as the sessions progressed (Figure 1B). Lever press data are shown in Figure 1C, with rats increasing their Active Lever presses over sessions and discriminating the fentanyl-paired Active Lever from the Inactive Lever, with main effects of Lever (*F*_1_,_27_ = 19.26, *p* < 0.001) and Session (*F*_9_,_243_ = 6.87, *p* < 0.001), and a Lever × Session interaction (*F*_9_,_243_ = 4.88, *p* < 0.001).

*Incubation testing*: To examine the involvement of BNST CRFR1 signaling in incubation of opioid craving, we gave all rats intra-BNST vehicle injections on Day 1, while on Day 30 we gave approximately half the rats intra-BNST vehicle and the other half intra-BNST R121919. During tests, there are no infusions earned, only cues earned on the same schedule of reinforcement as acquired during self-administration training. Cues earned data are shown in Figure 1D. We observed a main effect of Withdrawal Day (*F*_1_,_27_ = 15.51, *p* = 0.001) indicating rats earned more cues on Day 30 relative to Day 1, consistent with an incubation effect. We also observed a Withdrawal Day × Day 30 Treatment interaction (*F*_1_,_27_ = 9.50, *p* = 0.005). *Post hoc* analysis of cues earned revealed that intra-BNST vehicle treated rats earned more cues on Day 30 compared to Day 1 (*t*_12_ = −4.93, *p* < 0.001) while intra-BNST R121919 treated rats did not (*t*_14_ = −0.62, *p* > 0.05), suggesting incubation is prevented by treatment with the CRFR1 antagonist in the BNST.

Lever press data are shown in Figure 1E. We observed main effects of Lever (*F*_1_,_26_ = 35.46, *p* < 0.001) and Withdrawal Day (*F*_1_,_26_ = 7.29, *p* = 0.012) suggesting that while overall rats discriminated the Active Lever from Inactive Lever, they pressed more on withdrawal Day 30 for both Active and Inactive levers compared to Day 1. There was also a Withdrawal Day × Day 30 Treatment interaction (*F*_1_,_26_ = 5.21, *p* = 0.031) but no other main effects or other interactions (*F*’s < 1.4, *p*’s > 0.05). Because we only gave treatment on Withdrawal Day 30, we analyzed the timecourse of lever responding (and cues earned; see section “Supplementary Material”) on Day 30 using within-subject factors of Lever (Active and Inactive) and Bin (30, 60, and 90 min) and between-subject factor of Day 30 Treatment (vehicle, R121919) (Figure 1F). This revealed main effects of Lever (*F*_1_,_26_ = 46.21, *p* < 0.001), Bin (*F*_2_,_52_ = 10.52, *p* = 0.002) and a Lever × Bin × Treatment interaction (*F*_2_,_52_ = 5.40, *p* = 0.007), indicating the effect of treatment across bins, varies by lever. An analysis of Active Lever data revealed main effects of Bin (*F*_2_,_52_ = 6.92, *p* = 0.002) and Day 30 Treatment (*F*_1_,_26_ = 4.30, *p* = 0.049) but the interaction did not reach significance (*F*_2_,_52_ = 2.49, *p* = 0.093). These data suggest, relative to vehicle, intra-BNST R121919 treatment attenuates Active Lever pressing across the entire Day 30 test session. While the Bin × Day 30 treatment interaction was only trending toward significance, qualitatively, it is notable that the greatest difference between R121919 and vehicle treatment is observed in the final bin of the Day 30 test session. We confirmed active responding timecourse did not differ for prospective Day 30 treatment groups by examining the time course of Active Lever responding on Day 1 test (Supplementary Figure 1A), which showed main effect of Bin (*F*_2_,_52_ = 13.03, *p* < 0.001) but no other main effects or interactions (*F*’s < 0.26, *p*’s > 0.05). While analysis of Day 30 Inactive Lever data showed a Bin × Treatment interaction (*F*_2_,_52_ = 4.65, *p* = 0.014), the difference in first bin responding did not reach significance between treatment groups (*post hoc t* test *p* > 0.05). Altogether these data suggest the effect of intra-BNST R121919 was specific to Active Lever responding on Day 30 and was not due to differences between groups on Day 1.

### Experiment 2: Incubation of Fentanyl Craving in Opioid Dependence

**FIGURE 2:**
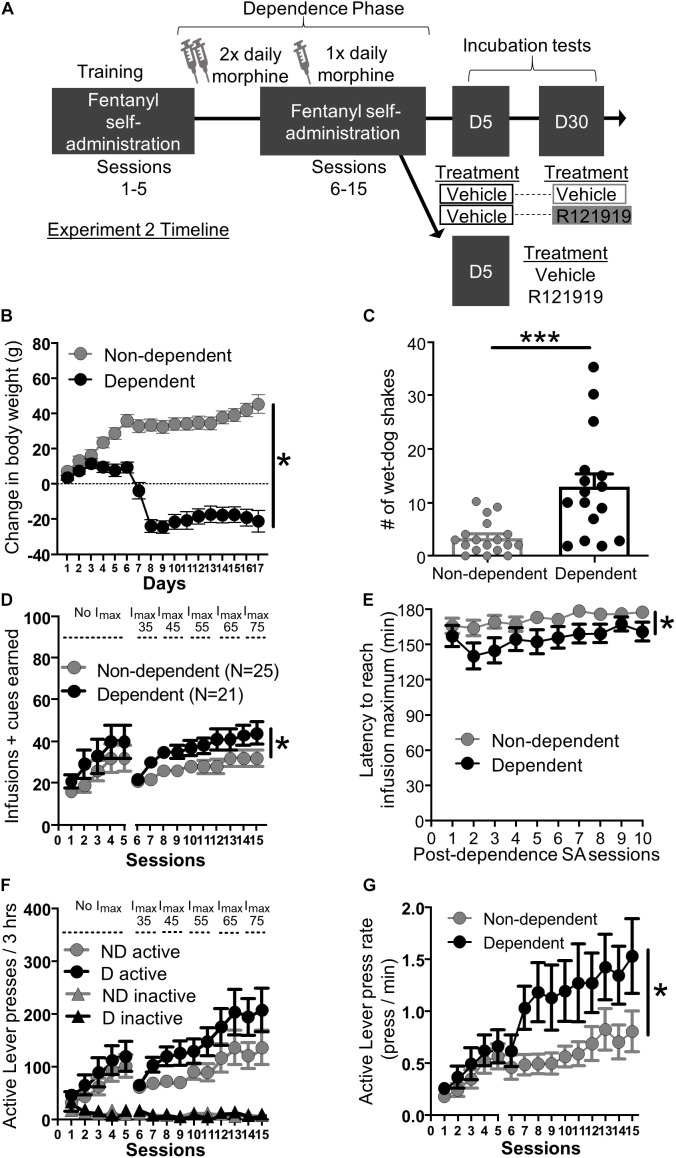
Experiment 2: Fentanyl self-administration training in opioid dependent versus non-dependent rats. **(A)** Experimental timeline: We trained rats to self-administer fentanyl for 5 days before inducing opioid dependence. Rats resumed self-administration for 10 more days while dependence was maintained. On forced abstinence Day 5 and Day 30, we measured lever responding under extinction conditions. **(B)** Change in body weight across days for opioid-dependent and non-dependent rats after start of dependence phase. **(C)** Number of wet-dog shakes for opioid-dependent and non-dependent rats. **(D)** Fentanyl infusions + cues earned across the 15 3 h self-administration sessions, sessions 1–5: before dependence and no infusion maximum criteria, 6–15: during dependence with infusion maximum criteria. **(E)** Latency to reach infusion maximum criteria in minutes. **(F)** Active/Inactive Lever pressing for dependent and non-dependent rats during training. **(G)** Active Lever press rate. *p < 0.05, main effect of dependence, ***p < 0.001, different from non-dependent. SA, Self-administration; ND, non-dependent; D, dependent. Data are mean ± SEM.

Experimental timeline is shown in Figure 2A. Briefly, we trained rats 3 h/day for 5 days in fentanyl self-administration before giving twice daily morphine/saline injections for 7 days. We then resumed daily self-administration training for 10 more days with once daily morphine/saline injection.

*Dependence measures*: To investigate the effectiveness of the morphine regimen to induce dependence in our rats, we compared somatic signs of opioid withdrawal between opioid-dependent and non-dependent rats. We examined change in body weight across training and dependence (Figure 2B) and wet dog shakes 24 h after their last self-administration training and morphine/saline injections (Figure 2C). While non-dependent rats showed weight gain, dependent rats lost weight across training during the dependence phase (Figure 2B). Repeated measures ANOVA on body weight including within-subject factor of Day and between-subject factor of Dependence yielded main effects of Day (*F*_16_,_464_ = 23.56, *p* < 0.001) and Dependence (*F*_1_,_29_ = 51.43, *p* < 0.001) as well as a Session × Dependence interaction (*F*_16_,_464_ = 82.66, *p* < 0.001). In addition, dependent rats also displayed more wet-dog shakes, 24 h after the last morphine injection, compared to non-dependent rats (Figure 2C; independent samples *t*-test *t*_32_ = 3.93, *p* < 0.001).

*Self-administration training*: We analyzed the training data using mixed ANOVAs including within-subject factor of Session (either Sessions 1–5; before dependence phase, or Sessions 6–15; during dependence phase) and between-subject factor of Dependence (dependent, non-dependent). The self-administration infusion + cues earned data is shown in Figure 2D; we observed a main effect of Session for both pre-dependence sessions 1–5 (*F*_4_,_176_ = 12.20, *p* < 0.001) and during dependence phase sessions 6–15 (*F*_9_,_396_ = 14.01, *p* < 0.001) but no Session × Dependence interactions for either Sessions 1–5 and 6–15 (*F*_4_,_176_ = 0.19, *p* > 0.05 and *F*_9_,_396_ = 1.34, *p* > 0.05 respectively). As described in section “Materials and Methods,” we set an infusion maximum (*I*_max_) for self-administration Sessions 6 through 10 in order to limit differences in fentanyl consumption/cue exposure between dependent and non-dependent rats. Despite this, we still observed a main effect of Dependence on infusions + cues earned during dependence phase Sessions 6–15 (*F*_1_,_44_ = 4.80, *p* = 0.034) but not pre-dependence phase (Sessions 1–5: *F*_1_,_44_ = 1.10, *p* > 0.05). Dependent rats reached *I*_max_ faster than non-dependent rats (Figure 2E). Mixed ANOVA on latency to reach *I*_max_ data including within-subject factor of Session and between-subject factor of Dependence revealed main effect of Session (*F*_9_,_396_ = 3.73, *p* < 0.001) and Dependence (*F*_1_,_44_ = 5.03, *p* = 0.030) but no interaction (*F*_9_,_396_ = 0.81, *p* > 0.05). Similar to infusions + cues earned, all rats increased their Active Lever responding over sessions and discriminated fentanyl-paired Active Lever from Inactive Lever (Figure 2F). For pre-dependence phase we ran a repeated measures ANOVA including within-subject factors of Lever (Active and Inactive) and Sessions (1–5) and between-subject factor of Dependence. The ANOVA yielded main effects of Lever (*F*_1_,_44_ = 28.00, *p* < 0.001), Session (*F*_4_,_176_ = 13.23, *p* < 0.001), and a Lever × Session interaction (*F*_4_,_45_ = 13.00, *p* < 0.001), but no other interactions (*F*’s < 0.42, *p*’s > 0.05). For the dependence phase self-administration sessions 6–15 we ran a repeated measures ANOVA with Lever (Active and Inactive) and Session (6–15) as within-subject factors and Dependence as between-subject factors. This resulted in a main effect of Session (*F*_9_,_396_ = 11.62, *p* < 0.001), Lever (*F*_1_,_44_ = 62.52, *p* < 0.001), and a significant Lever × Session interaction (*F*_9_,_396_ = 11.82, *p* < 0.001) but no other interactions (*F*’s < 2.20, *p*’s > 0.05). While there was no main effect of Dependence on lever presses during dependence phase self-administration sessions (Sessions 6–15: *F*_1_,_44_ = 2.69, *p* > 0.05), dependent rats pressed the fentanyl-paired Active Lever more vigorously compared to non-dependent rats (Figure 2G). Active Lever press rate $\left(\frac{A\,c\,t\,i\,v\,e\,\,L\,e\,v\,e\,r\,\,p\,r\,e\,s\,s\,e\,s}{L\,a\,t\,e\,n\,c\,y\,\,t\,o\,\,r\,e\,a\,c\,h\,\,I_{\max}-L\,a\,t\,e\,n\,c\,y\,\,t\,o\,\,f\,i\,r\,s\,t\,\,p\,r\,e\,s\,s}\right)$ from Sessions 1–5 to 6–15 was analyzed using mixed ANOVA with Session as a within-subject factor and Dependence as a between-subject factor. We found a main effect of Session (1–5: *F*_4_,_176_ = 18.30, *p* < 0.001, 6–15: *F*_9_,_396_ = 4.01, *p* < 0.001), and a main effect of Dependence on Sessions 6–15 (*F*_1_,_44_ = 5.29, *p* = 0.026), but not on Session 1–5 (*F*_1_,_44_ = 0.62, *p* > 0.05) and no Session × Dependence interactions during either phase (*F*’s < 0.7, *p*’s > 0.5).

*Testing*: After the dependence phase of self-administration, we split the rats into two groups: (1) a protracted withdrawal day treatment group tested both on Day 5 (vehicle/vehicle) and Day 30 (vehicle/R121919) to examine the effects of CRFR1 antagonism on fentanyl seeking during incubation and (2) an acute withdrawal day treatment group tested only on Day 5 (vehicle/R121919) to examine the effects of CRFR1 antagonism on fentanyl seeking during acute withdrawal.

*Protracted withdrawal (Day 5 vs Day 30 test)*: All (dependent and non-dependent) rats received intra-BNST vehicle injections on Day 5, while half from each group received intra-BNST vehicle and the other half intra-BNST R121919 on Day 30. To our surprise, we did not observe the expected time-dependent increase, or incubation, for cues earned between Day 5 and Day 30. Cues earned data are shown in Supplementary Figure 2A. Instead, we observed the opposite, a reduction in number of cues earned on Day 30 relative to Day 5 across dependence and treatment groups. The ANOVA yielded a main effect of withdrawal day (*F*_1_,_12_ = 8.80, *p* = 0.012) indicating reduction in cues earned between Day 5 and day 30. This could be driven, in part by treatment effects on day 30, but because the number of subjects per group was low, we were unable to detect differences between Dependence groups or notable effects of Day 30 Treatment on the low levels of responding present on Day 30 (Dependence, Day 30 Treatment main effects and interactions all *F*’s < 3.12, *p*’s > 0.05). Despite this, we observed large effect sizes for cues earned between Day 5 and Day 30 tests (Cohen’s *d* ≥ 1.28) for rats treated with intra-BNST R121219 on Day 30, but only small effect sizes for rats treated with intra-BNST vehicle on Day 30 (Cohen’s *d* 0–0.45), see section “Supplementary Results” for more detail. Together, this suggests that despite reduced responding on Day 30, CRFR1 antagonist likely acts to decrease cues earned during protracted withdrawal independent of dependence condition.

**FIGURE 3:**
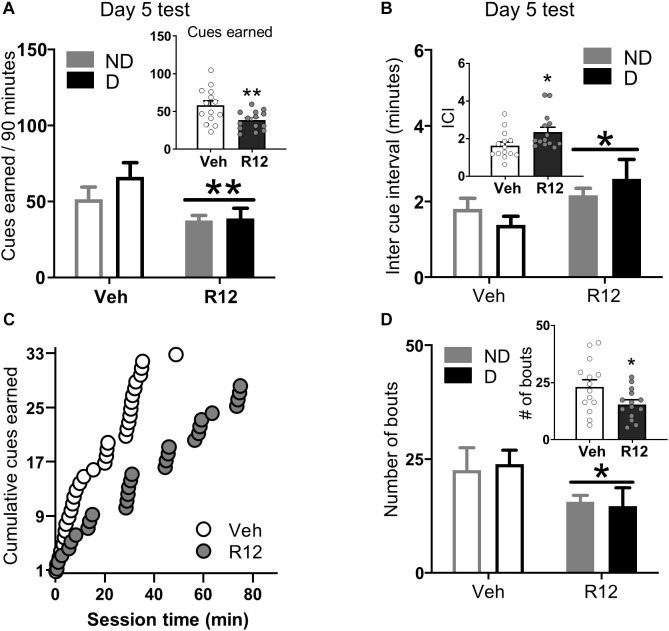
Experiment 2: BNST CRFR1 antagonist effect on fentanyl seeking during acute withdrawal (Day 5) test. **(A)** Day 5 incubation test data showing cues earned on FR1, 20 s to schedule. **(B)** Inter cue interval (ICI), the time between presses that result in cues. **(C)** Cumulative cues earned pattern of a representative pair of vehicle ($\bar{X}$ = 1.52) and R121919 ($\bar{X}$ = 2.75) injected rat. **(D)** Number of bouts. A bout is defined as two or more presses for which the interval between successive presses did not exceed 20 s. *p < 0.05, **p < 0.01, main effect of treatment. All the inset graphs indicate mean ± SEM when collapsed across dependence. ND, non-dependent; D, dependent. Data are mean ± SEM.

*Acute Withdrawal (Day 5 test)*: To understand the role of BNST CRFR1 signaling for driving fentanyl seeking after acute withdrawal, we gave approximately half of the rats from each dependence group intra-BNST vehicle and the other half intra-BNST R121919 on Day 5. Cues earned data for acute withdrawal Day 5 are shown in Figure 3A. Rats injected with intra-BNST R121919 earned fewer cues on Day 5 test compared to vehicle injected rats (Figure 3A). The ANOVA on cues earned resulted in a main effect of Day 5 Treatment (*F*_1_,_24_ = 8.36, *p* = 0.008), but no main effect of Dependence (*F*_1_,_24_ = 1.28, *p* > 0.05) or a Day 5 Treatment × Dependence interaction (*F*_1_,_24_ = 0.89, *p* > 0.05). When we analyzed the pattern of pressing resulting in cue delivery, we found that more time elapsed between cue presentations for rats injected with intra-BNST R121919 compared to the intra-BNST vehicle injected rats (Figure 3B). An ANOVA on average inter cue interval (ICI) data revealed a main effect of Day 5 Treatment (*F*_1_,_24_ = 6.18, *p* = 0.020) but no main effect of Dependence or a Day 5 Treatment × Dependence interaction (*F*’s < 2.40, *p* > 0.05). Figure 3C shows cumulative cues earned during a representative session from an intra-BNST vehicle and an R121919 injected rat. Qualitatively, this exemplifies the longer time elapsed between reinforced presses in R121919 relative to vehicle treated rats.

We performed an analysis of lever press bouts to determine whether the CRFR1 antagonist affected the way in which rats engaged in drug seeking during the test session. Compared to vehicle treated rats, intra-BNST R121919 treatment decreased the number of press bouts (Figure 3D). The ANOVA on number of press bouts resulted in a main effect of Day 5 Treatment (*F*_1_,_24_ = 4.70, *p* = 0.040), but no other main effects or interactions *F*’s < 0.10, *p*’s > 0.05. The ANOVA on inter-bout-interval data resulted in a marginally significant main effect of Day 5 Treatment (*F*_1_,_24_ = 4.01, *p* = 0.054; Supplementary Figure 3C) but no other main effects or interactions (*F*’s < 1.94, *p*’s > 0.05). R121919 treatment did not affect bout size or bout duration (Supplementary Figures 3A,B; *F*’s < 1.77, *p*’s > 0.5). The overall lever press, press vigor, and time out response data followed similar patterns (Supplementary Material and Supplementary Figures 3D–F). Altogether, these data show BNST CRFR1 antagonism limits rats from re-engaging in opioid seeking during test, resulting in less fentanyl-associated conditioned reinforcement.

*Opioid demand*: Finally, to confirm intra-BNST R121919 did not affect opioid consumption or ability to lever press we trained the acute withdrawal rats to self-administer short acting opioid, remifentanil in opioid demand threshold procedure. We compared remifentanil consumption at low cost (*Q*_0_), demand elasticity (*α*), total remifentanil consumption, total Active Lever presses, and Active Lever presses in the last bin when the rats were injected with intra-BNST vehicle versus R121919. We found no significant difference in any of these measures between vehicle treated or R121919 treated conditions suggesting that intra-BNST R121919 injection doesn’t preclude animals from lever pressing at high levels (∼400 presses/session) or consuming opioid drugs of abuse (see section “Supplementary Results” and Supplementary Figure 4).

**FIGURE 4:**
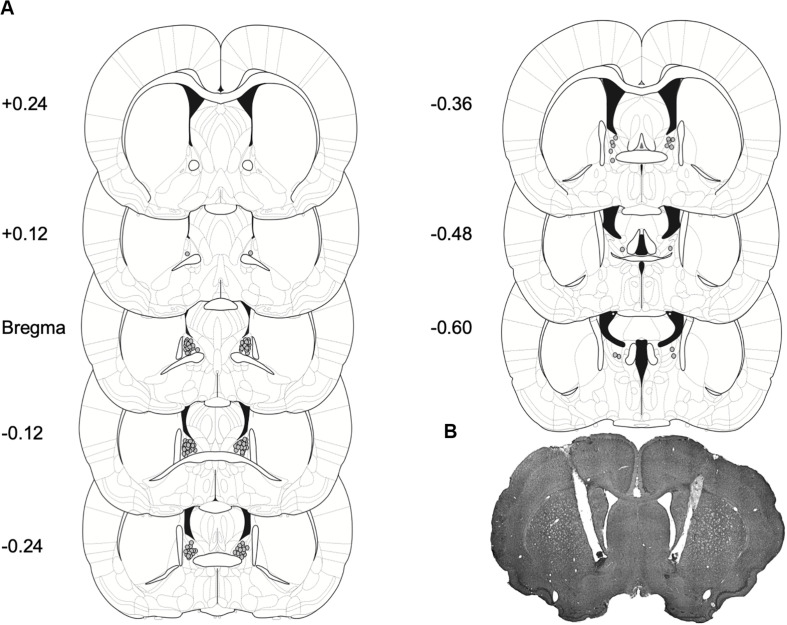
Placement for injector tips in rats with BNST cannulae implants. **(A)** Gray dots represent sites of R121919 infusions on coronal sections as a distance from Bregma (mm). Drawings are adapted from Paxinos and Watson (2007). **(B)** A representative brain section depicting cannulae placement in the BNST.

*Histological verification*: Cannula placements for Experiments 1 and 2 are shown in Figure 4A. A majority of the placements were observed on the anterior to posterior axis between Bregma and −0.24 to Bregma, as summarized in Figure 4A and in representative image Figure 4B.

## Discussion

Here, we found BNST CRFR1 receptor antagonist reduced incubation of fentanyl craving after a month of forced abstinence. While control rats display incubated cue-reinforced fentanyl seeking after protracted withdrawal, rats injected with the CRFR1 antagonist R121919 did not display this time dependent increase in cue-reinforced opioid seeking. Next, we sought to determine whether opioid dependence enhanced incubation and determine the role of BNST CRFR1 signaling in opioid seeking. Again, R121919 attenuated cue-reinforced opioid seeking after an acute period of withdrawal in both dependent and non-dependent rats. Closer analysis revealed CRFR1 receptor antagonist acts to prevent re-engagement with the cue-reinforced lever pressing after periods of disengagement during test. Together, these results suggest a critical role of BNST CRFR1 receptors in cue reinforced opioid seeking after periods of forced abstinence and extend previous findings on the important role of CRFR system in opioid addiction (Contarino and Papaleo, 2005; Papaleo et al., 2007; Greenwell et al., 2009; Logrip et al., 2011; Roberto et al., 2017; Reiner et al., 2019).

### Methodological Considerations

In the present study, we used the same schedule of reinforcement for training and test, maintaining the 20 s timeout period between cue-reinforcement at test. Maintaining the same reinforcement schedule during testing enabled us to examine patterns of drug seeking based on previously learned cue reinforcement contingencies in the absence of the drug, instead of newly acquired cue reinforcement contingencies. Further, it provided us with a deeper understanding of the role of BNST CRFR1 receptors, as R121919 treatment increased the interval between reinforced presses and reduced the amount of fentanyl-associated conditioned reinforcement during test.

Here, we trained rats daily for 3 h to self-administer fentanyl, different from many incubation studies that train rats for 6 h or more (Reiner et al., 2019; Grimm, 2020). Even with limited training, we observed escalation of drug intake in both experiments (Figures 1, 2). Moreover, we used a mixed within/between-subject design to compare responding on Day 1 versus Day 30 (within factor) in control and treated (between factor) rats, as opposed to a completely between subject design. We observed both incubation and treatment effects, demonstrating incubation of craving studies are accessible using limited access self-administration and mixed experimental design.

Because we did not infuse R121919 intra-BNST on Day 1 we cannot rule out the possibility that R121919 may have affected responding for cues prior to incubation. We don’t expect this is the case because we found no difference in responding between vehicle and R121919 treatment in the last bin of demand sessions when drug dose is extremely low and non-reinforcing (Supplementary Figure 4E). Notably, in this last bin of remifentanil demand, any prior infusions have quickly cleared from the system and similar to Day 1 tests, cues alone are what reinforces responding during this time bin. While this is not an optimal representation of Day 1 responding, we tentatively conclude that in a non-incubated state, intra-BNST infusion of R121919 doesn’t affect rats’ lever pressing for cues. Future experiments with Day 1 infusion of intra-BNST R121919 are necessary to confirm this conjecture.

An unexpected finding using the mixed within/between design in Experiment 2 was that rats responded less on Day 30 compared to Day 5. This could be due to several factors. First, rats responded at high levels during the Day 5 test (more than two times higher than Experiment 1 Day 1 test), leading to more extinction experience on Day 5. This may have resulted in enhanced extinction learning that interfered with incubation and reduced responding on Day 30. Another possibility is that by Day 5, incubation of fentanyl craving might have had already occurred resulting in the high level responding we observed in the first test. Indeed, there is evidence for incubation of opioid craving as early as 6 days (Shalev et al., 2001). Incubation data classically follow an inverted U-shaped curve, with low responding on day 1, maximal responding occurring between 6 and 30 days that returns to day 1 levels after 2 months of withdrawal from heroin (Shalev et al., 2001; Pickens et al., 2011). Finally, in experiment 2, we incorporated a dependence phase and extended self-administration experience well beyond the typical ten days of opioid experience. These factors could have influenced the level of responding we observed on Day 30, which could, in part, be due to testing on the backside of the incubation curve. Lack of incubation for discrete cues has been observed for other reinforcers, including alcohol seeking after abstinence (Jupp et al., 2011) and for context induced incubation of methamphetamine seeking (Adhikary et al., 2017). While we failed to observe incubation for discrete fentanyl associated cues in Experiment 2, the evidence for incubation on Experiment 1 suggests that the lack of incubation in Experiment 2 is a procedural issue and not lack of incubation of opioid craving which is well established pre-clinically (Reiner et al., 2019).

Relative to control manipulations, we observed substantially lower levels of cue-reinforced responding with BNST CRFR1 antagonism on Day 30, suggesting a role for this system in driving opioid seeking after protracted withdrawal. Despite this, future studies are needed to confirm whether BNST CRFR1 signaling is involved in protracted withdrawal after opioid dependence. An important consideration for future studies is to strike a balance between testing early enough in acute withdrawal to capture the front end of the incubation curve, while still avoiding the most severe opioid withdrawal symptoms that could interfere with lever pressing.

### BNST CRFR1 Receptors in Relapse and Dependence

Several studies have implicated CRF action in the BNST in drug addiction related behaviors. Intra-BNST CRF antagonists block stress-induced reinstatement of morphine conditioned place preference (Wang et al., 2006) and cocaine reinstatement following foot shock (Erb and Stewart, 1999), whereas intra-BNST CRF infusions promote stress induced cocaine reinstatement (Erb and Stewart, 1999; Mantsch et al., 2016). The CeA is one source for CRF in the BNST that is heavily implicated in addiction related behaviors (Mantsch et al., 2016). A study using pharmacological inactivation of the CRF pathway asymmetrically inactivating the CeA and antagonizing BNST CRF receptors showed that this pathway drives footshock induced reinstatement of cocaine seeking (Erb et al., 2001). More recently, optogenetic silencing of the CeA → BNST CRF circuitry reduced alcohol seeking only in alcohol-dependent rats (de Guglielmo et al., 2019). This study went on to demonstrate that BNST CRFR1 is the primary site of action for this effect. While there is evidence that BNST neurons can synthesize CRF locally within the BNST (Shepard et al., 2006), it is not clear whether local action of BNST synthesized CRF plays a role in addiction related behaviors. We predict that the effect of BNST CRFR1 antagonism on cue reinforced fentanyl seeking observed here is likely due to blocking the action of CRF released from CeA terminals. Future opioid incubation studies using projection specific manipulations to target CeA → BNST CRF transmission could test this prediction.

The BNST CRFR1 system is previously implicated in opioid withdrawal (Nakagawa et al., 2005; Greenwell et al., 2009; Luster et al., 2020). In the present study, we investigated the role of BNST CRFR1 in driving the time dependent increase in cue induced opioid seeking, attempting to understand how opioid dependence and acute withdrawal states influence subsequent self-administration and incubation of opioid craving. During self-administration we observed that dependent rats consumed more fentanyl and were exposed to more fentanyl-associated cues than non-dependent rats. Despite this, we did not see a difference in opioid seeking between dependent and non-dependent rats during acute or protracted withdrawal tests. Further, the CRFR1 receptor antagonist, R121919, in the BNST did not differentially affect the behavior of dependent and non-dependent rats during these tests. Systemic injections of R121919 dose dependently decrease heroin seeking in heroin-dependent rats (Greenwell et al., 2009). In that study rats were injected systemically with R121919 while still having access to opioids. In contrast, in our study we injected rats with R121919 intra-BNST when rats had no access to fentanyl but only fentanyl associated cues. R121919 attenuated cue-reinforced presses both in the protracted withdrawal incubation test and in the acute withdrawal test for both dependent and non-dependent rats. Notably, our tests were conducted in rats with a history of dependence, tested after the dependence phase had ended, which may have limited our ability to detect differences in treatment effects between dependence groups. When we trained rats in a within session economic demand paradigm to self-administer remifentanil, we found no difference between intra-BNST vehicle or R121919 treated conditions for remifentanil consumption at low price, total consumption, or in total Active Lever presses. This suggests that BNST CRFR1 receptors are uniquely involved in cue-reinforced opioid seeking after forced abstinence, but not in ongoing opioid consumption.

Further, intra-BNST R121919 increased the interval between reinforced presses, suggesting the antagonist promotes disengagement from opioid seeking. There could be several possibilities by which R121919 could be limiting engagement in cue-reinforced opioid seeking. Mechanistically, CRF and CRFR1 receptor expression is altered in the amygdala and extended amygdala after chronic drug use and withdrawal (Maj et al., 2003; Sommer et al., 2008; Roberto et al., 2017). We speculate that R121919 could be attenuating the enhanced transmission at BNST CRFR1 receptors to promote disengagement from opioid seeking. Psychologically, CRF action promotes aversive states that motivate drug-seeking behaviors (Roberto et al., 2017). If we had observed stronger effects of CRFR1 antagonism in dependent rats we may have speculated that R121919 alleviated conditioned aversive states, however, we did not see evidence for this. Instead we expect intra-BNST R121919 acts to diminish the incentive properties of fentanyl associated cues to promote disengagement from opioid seeking. Future experiments are needed to disentangle these psychological constructs and identify the precise underlying mechanisms.

### Limitations and Conclusion

Extrahypothalamic CRFR1 system antagonism has been shown to exacerbate somatic signs of spontaneous opioid withdrawal (Papaleo et al., 2007) while it attenuates somatic signs of naltrexone-induced precipitated opioid withdrawal (Iredale et al., 2000). Here, we did not determine if intra-BNST R121919 exacerbates or attenuates somatic signs of opioid withdrawal. This could be of interest in future studies. In addition, we used only male rats in this study which is an important limitation as it has been shown that a subpopulation of females is more sensitive to CRF-induced reinstatement of cocaine seeking compared to males (Buffalari et al., 2012). While there is limited evidence for sex differences in incubation of fentanyl seeking (Reiner et al., 2020), evidence for BNST-mediated sex differences in opioid withdrawal (Luster et al., 2020) encourage future studies investigating sex differences in BNST CRFR1 action. We used a single dose of R121919 (1 μg/hemisphere) based on a prior study (Pomrenze et al., 2019) to test the role of BNST CRFR1 receptors in fentanyl craving. There is evidence for CRFR1 antagonist reducing frustration stress-induced binge like palatable food consumption with dose as low as 25 ng in the BNST (Micioni et al., 2014). Future studies with multiple doses of R121919 are needed to test if lower doses of the antagonist are sufficient to reduce incubation of opioid craving. Our study bolsters previous findings on the importance of CRFR1 in addiction related behaviors. We provide evidence of the importance of BNST CRFR1 signaling for driving cue-reinforced opioid seeking after periods of forced abstinence and extend our understanding of this system in driving specific facets of opioid incubation.

## Data Availability Statement

All datasets presented in this study are included in the article/Supplementary Material.

## Ethics Statement

The animal study was reviewed and approved by University of Maryland Institutional Animal Care and Use Committee.

## Author Contributions

DC and UG conceived the project, designed the experiments, interpreted the data, and wrote the manuscript. UG and DM acquired the data and analyzed the data. All authors contributed to manuscript revision, read, and approved the submitted version.

## Conflict of Interest

The authors declare that the research was conducted in the absence of any commercial or financial relationships that could be construed as a potential conflict of interest.
