# Corrigendum: Role of BNST CRFR1 Receptors in Incubation of Fentanyl Seeking

**DOI:** 10.3389/fnbeh.2021.660759

**Published:** 2021-05-14

**Authors:** Utsav Gyawali, David A. Martin, Agnieszka Sulima, Kenner C. Rice, Donna J. Calu

**Affiliations:** ^1^Program in Neuroscience, School of Medicine, University of Maryland, Baltimore, Baltimore, MD, United States; ^2^Department of Anatomy and Neurobiology, School of Medicine, University of Maryland, Baltimore, Baltimore, MD, United States; ^3^Intramural Research Program, National Institute on Drug Abuse, National Institute on Alcohol Abuse and Alcoholism, Baltimore, MD, United States

**Keywords:** incubation, opioid dependence, withdrawal, CRF, BNST

In the original article, there were mistakes in Figures 2 and 3, and **Supplementary Figure 3**, as published. The lines in Figure 2C, Figures 3A,B and **D**, and **Supplementary Figure 3**, were erroneously connecting the individual data points and have been removed. The individual data is still represented in these figures and is identical to the published data but is no longer connected with lines. The corrected Figure 2, Figure 3, and **Supplementary Figure 3** appear below.

**Figure 2:**
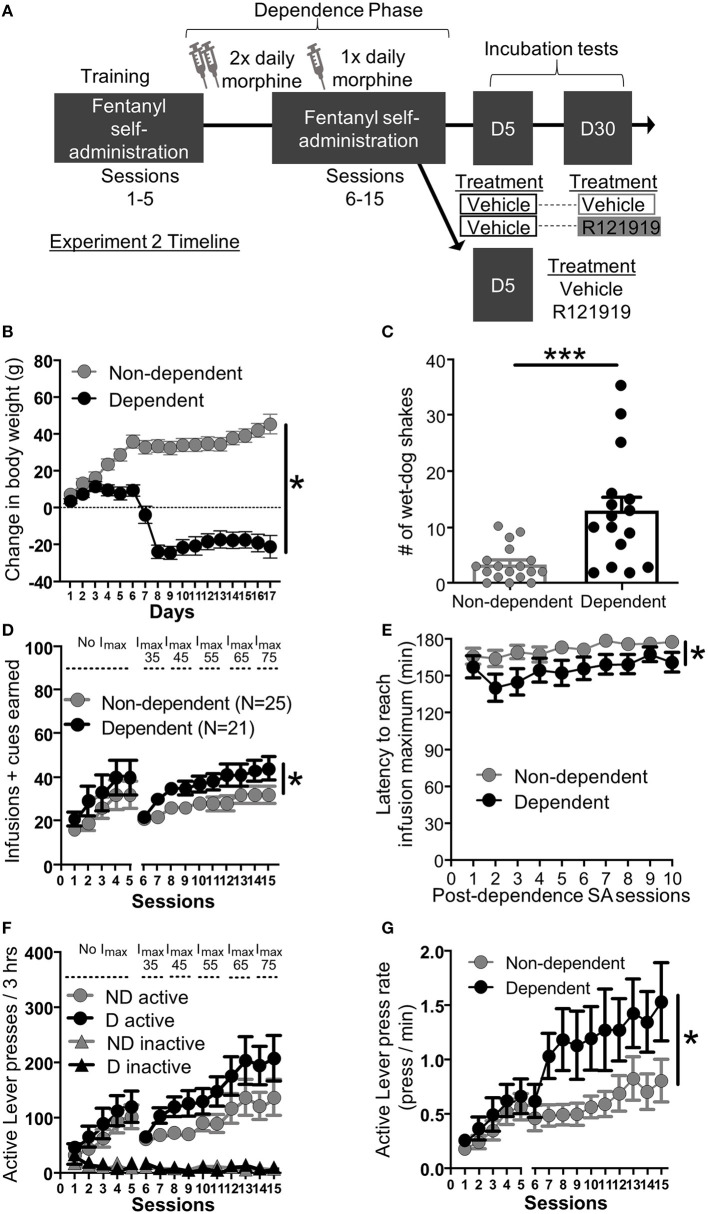
Experiment 2: Fentanyl self-administration training in opioid dependent versus non-dependent rats. **(A)** Experimental timeline: We trained rats to self-administer fentanyl for 5 days before inducing opioid dependence. Rats resumed self-administration for 10 more days while dependence was maintained. On forced abstinence Day 5 and Day 30, we measured lever responding under extinction conditions. **(B)** Change in body weight across days for opioid-dependent and non-dependent rats after start of dependence phase. **(C)** Number of wet-dog shakes for opioid-dependent and non-dependent rats. **(D)** Fentanyl infusions + cues earned across the 15 3 h self-administration sessions, sessions 1–5: before dependence and no infusion maximum criteria, 6–15: during dependence with infusion maximum criteria. **(E)** Latency to reach infusion maximum criteria in minutes. **(F)** Active/Inactive Lever pressing for dependent and non-dependent rats during training. **(G)** Active Lever press rate. *p < 0.05, main effect of dependence, ***p < 0.001, different from non-dependent. SA, Self-administration; ND, non-dependent; D, dependent. Data are mean ± SEM.

**Figure 3:**
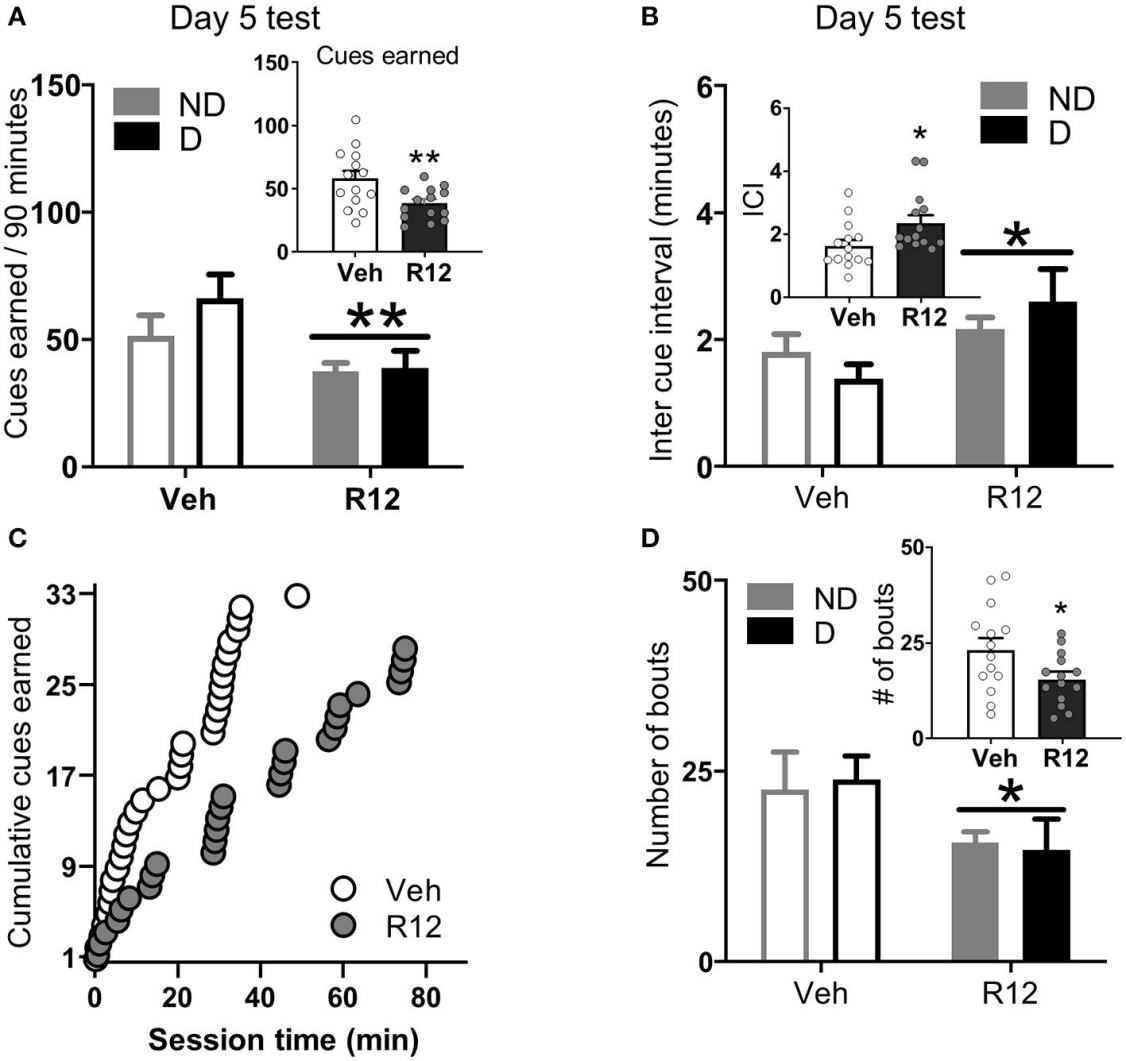
Experiment 2: BNST CRFR1 antagonist effect on fentanyl seeking during acute withdrawal (Day 5) test. **(A)** Day 5 incubation test data showing cues earned on FR1, 20 s to schedule. **(B)** Inter cue interval (ICI), the time between presses that result in cues. **(C)** Cumulative cues earned pattern of a representative pair of vehicle ($\bar{\text{X}}$ = 1.52) and R121919 ($\bar{\text{X}}$ = 2.75) injected rat. **(D)** Number of bouts. A bout is defined as two or more presses for which the interval between successive presses did not exceed 20 s. *p < 0.05, **p < 0.01, main effect of treatment. All the inset graphs indicate mean ± SEM when collapsed across dependence. ND, non-dependent; D, dependent. Data are mean ± SEM.

**Supplementary Figure 3** | **(A)** Bout size (number of presses per bout). **(B)** Bout duration (time from first to last press in a bout). **(C)** Inter bout interval, the time between last press in bout and first press in next bout. **(D)** Day 5 Incubation test data showing total lever presses (Active and Inactive). **(E)** Active lever press rate. **(F)** Time out responding (*Active Lever presses-cues earned*). All the inset graphs indicate mean ± SEM when collapsed across dependence. Veh = Vehicle, R12 = R121919, ND = Non-dependent. D = Dependent. Data are mean ± SEM.

Additionally, in the original article, there was an error related to testing schedules used in prior incubation studies that we misinterpreted. A correction has been made by removal of one statement in *Materials and Methods, Surgery, Day 1, 5 or 30 extinction testing*. The corrected paragraph is shown below.

*Day 1, 5 or 30 extinction testing:* We started the 90-min extinction tests 15 min after intracranial injections. For experiment 1, rats (*n* = 28) completed the extinction test on Day 1 and Day 30 while for experiment 2, rats completed the extinction test either on Day 5 only (*n* = 28) or Day 5 and Day 30 (*n* = 16). All extinction sessions began with extension of Active and Inactive Levers and illumination of red house light which remained on for the duration of the session. Presses on the Active Lever no longer resulted in drug infusions, but still resulted in contingent tone-light cue on the same FR1 20 s time out schedule of reinforcement used during training. Maintaining the same reinforcement schedule during testing enables us to examine patterns of drug seeking based on previously learned cue reinforcement contingencies in the absence of the drug, instead of newly acquired cue reinforcement contingencies in the absence of the drug. We recorded number of active presses for all experiments as well as the timestamp of each active press during day 5 test. Presses on the Inactive Lever were recorded but had no consequences.

A correction has also been made to *Discussion, Methodological Considerations, Paragraph 1*. The corrected paragraph is shown below.

**Methodological Considerations**

In the present study, we used the same schedule of reinforcement for training and test, maintaining the 20 s timeout period between cue-reinforcement at test. Maintaining the same reinforcement schedule during testing enabled us to examine patterns of drug seeking based on previously learned cue reinforcement contingencies in the absence of the drug, instead of newly acquired cue reinforcement contingencies. Further, it provided us with a deeper understanding of the role of BNST CRFR1 receptors, as R121919 treatment increased the interval between reinforced presses and reduced the amount of fentanyl-associated conditioned reinforcement during test.

The authors apologize for these errors and state that they do not change the scientific conclusions of the article in any way. The original article has been updated.

