# Supplementary figures and images for: Role of BNST CRFR1 Receptors in Incubation of Fentanyl Seeking
Source: Front Behav Neurosci. 2020 Aug 28;14:153. doi: 10.3389/fnbeh.2020.00153 (PMC7493668; doi:10.3389/fnbeh.2020.00153)

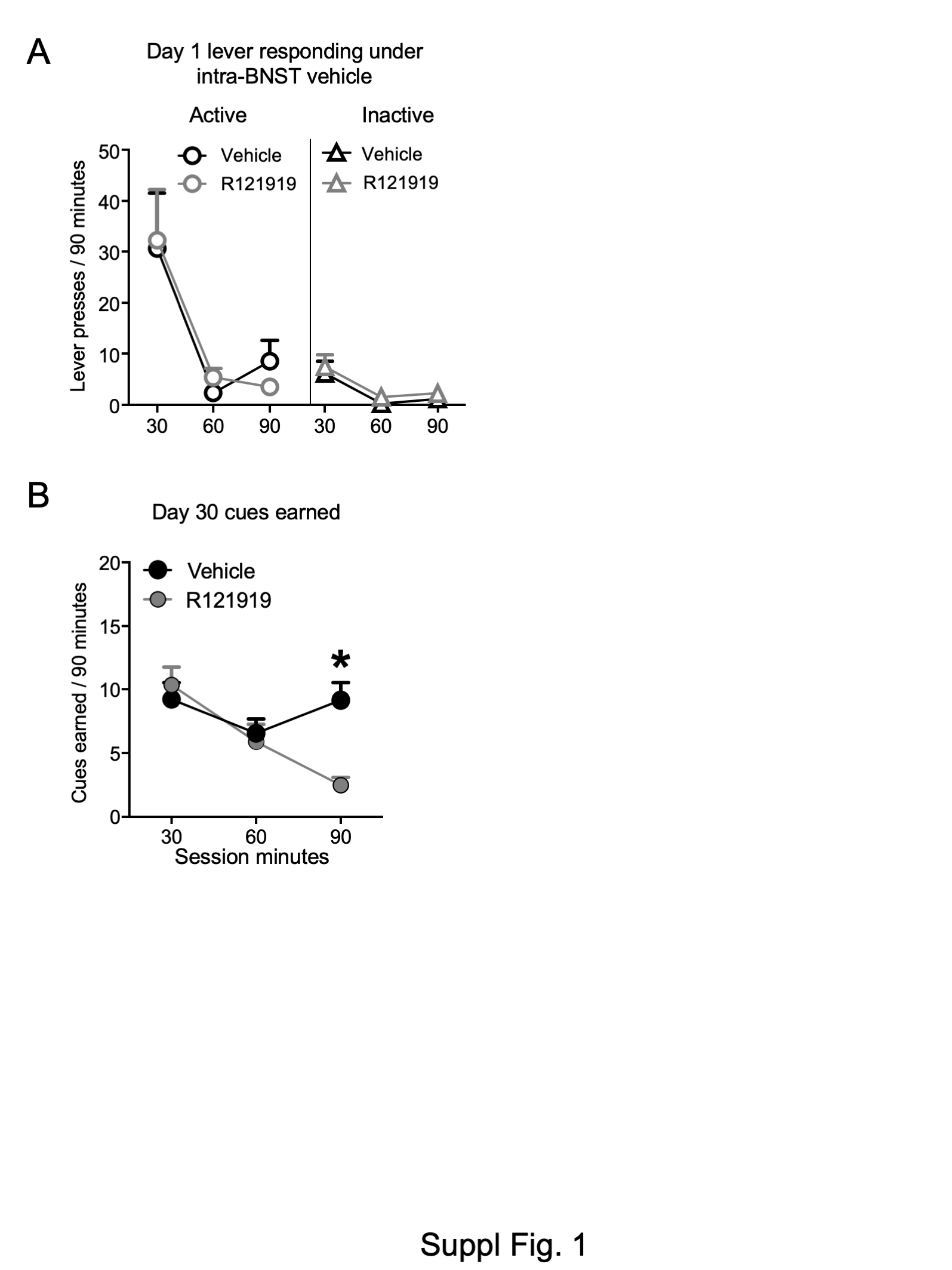

Supplement: Supplementary file 1 [file Image_1.TIFF]

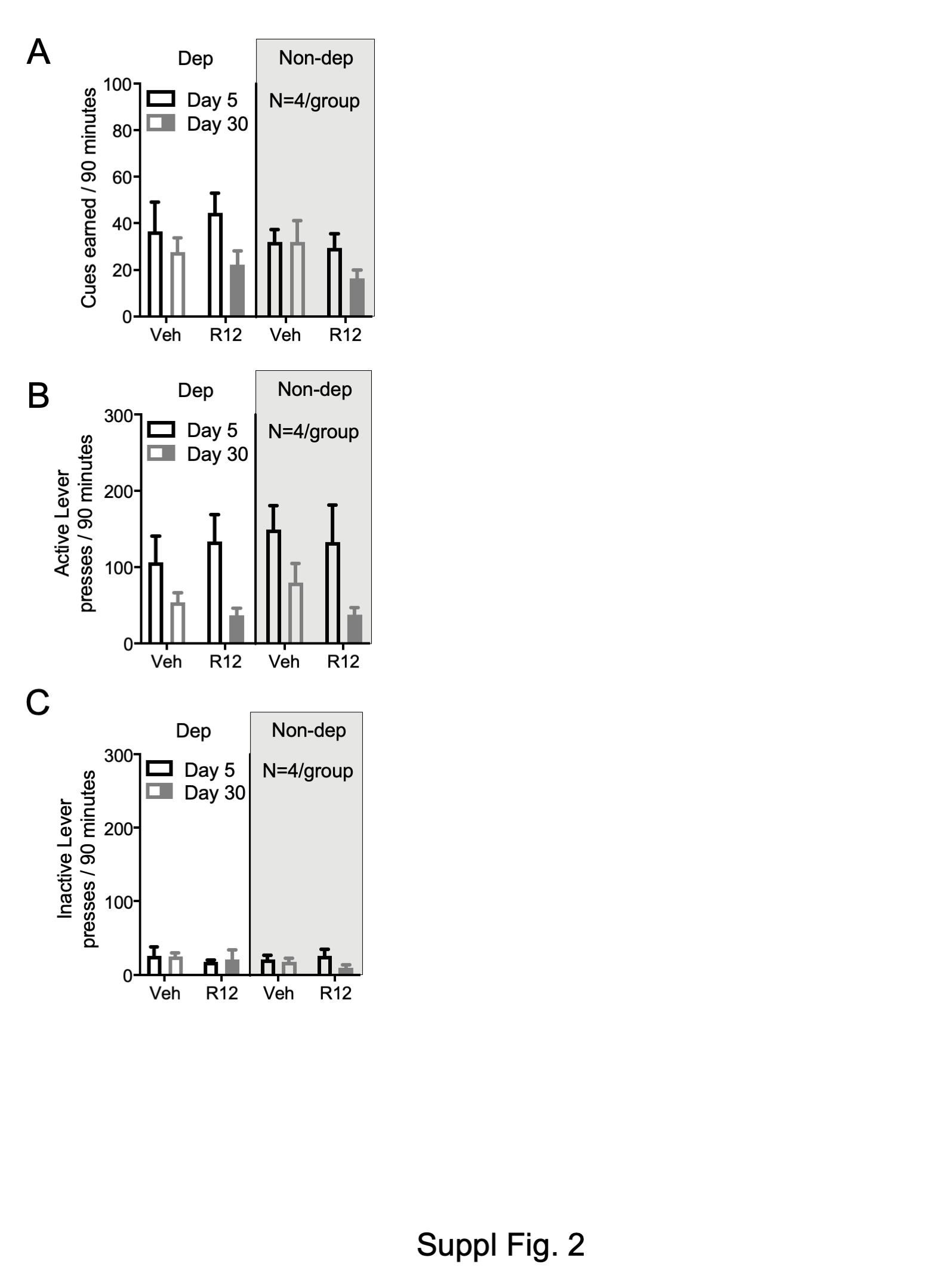

Supplement: Supplementary file 2 [file Image_2.TIFF]

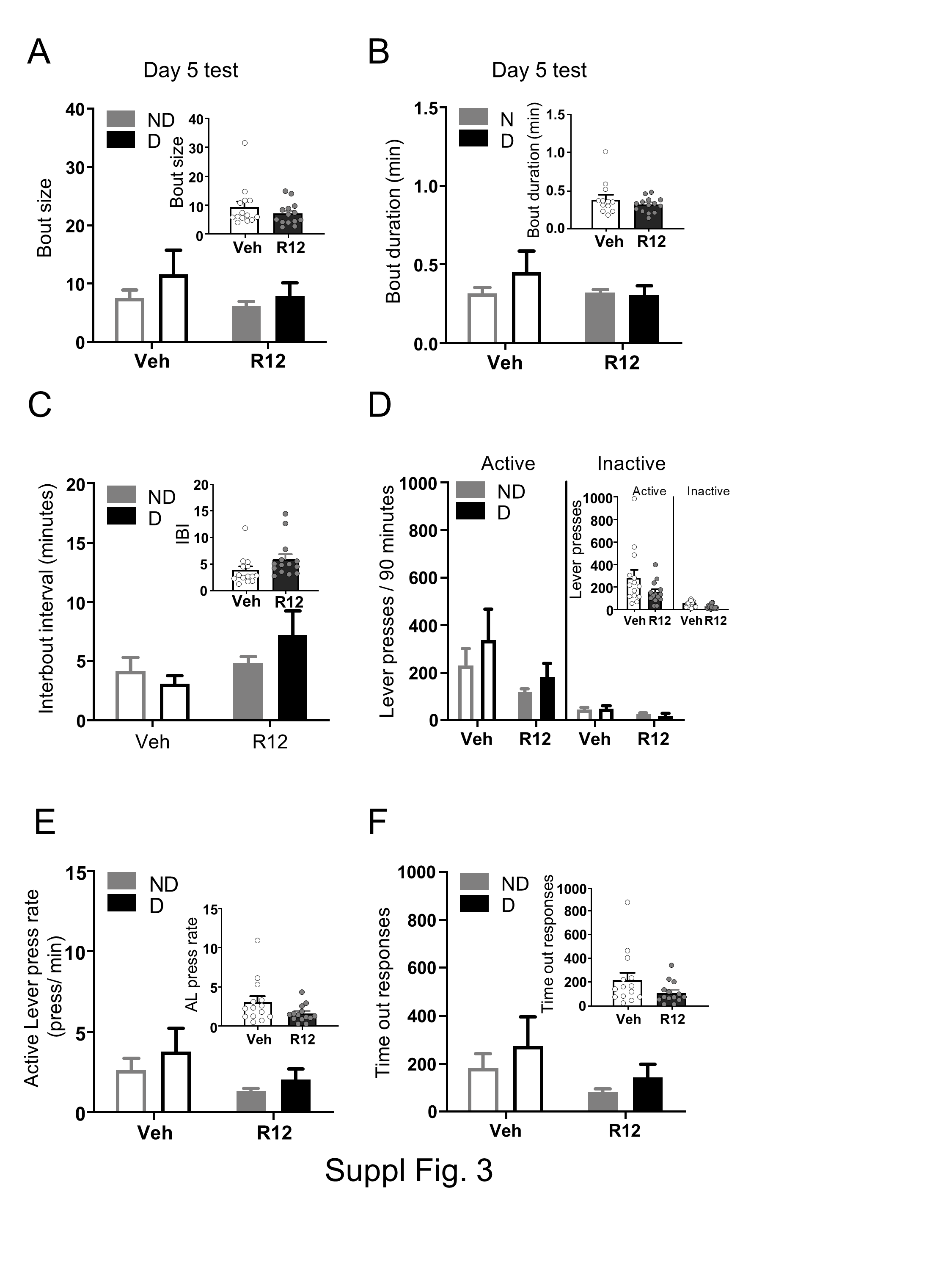

Supplement: Supplementary file 3 [file Image_3.tif]

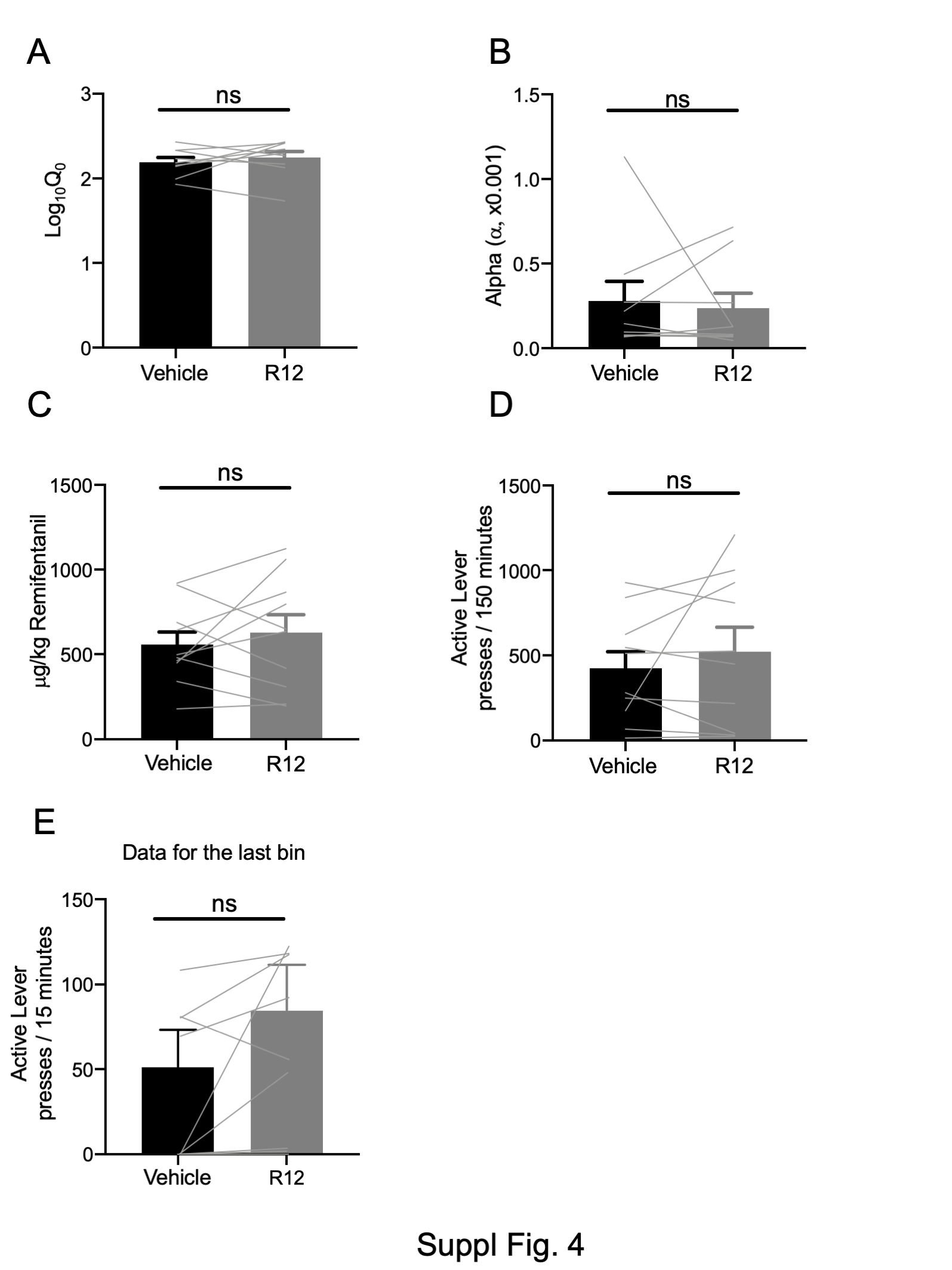

Supplement: Supplementary file 4 [file Image_4.TIFF]
