# Supplementary material for: Role of BNST CRFR1 Receptors in Incubation of Fentanyl Seeking
Source: Front Behav Neurosci. 2020 Aug 28;14:153. doi: 10.3389/fnbeh.2020.00153 (PMC7493668; doi:10.3389/fnbeh.2020.00153)
Supplement: Supplementary file 5 [file Table_1.docx]

**Supplementary Results**

*Experiment 1*

When we analyze terminal responding (Sessions 8-10) for the two Exp. 1 cohorts during self-administration we saw no differences between cohorts for infusions + cues earned or lever pressing. A repeated measures ANOVA with Sessions (8-10) as the within subject factor and cohort (1, 2) as between subject factor, we observed no main effectsor any interactions (*F*’s < 0.7, p’s > 0.05) for infusions + cues. Further, a repeated measure ANOVA with Lever (Active, Inactive) and Sessions (8-10) as within subject factors and cohort (1,2) as between subject factors showed a main effect of Lever (*F*_1,26_= 13.81, p<0.001), and Session (*F*_2,52_= 3.27, p=0.046) but no main effect of cohort or other interactions (*F*’s <3.02, p’s > 0.05). Hence, we pooled the two cohort’s data together.

We analyzed the timecoursefor cues earned on Day 30 using within-subject factors of Bin (30, 60, 90 min) and between-subject factor of Day 30 Treatment (vehicle, R121919) (Supplementary Fig. 1B). This resulted in main effect of Bin (*F*_2,52_ = 8.76, p=0.001)and a Bin x Treatment interaction (*F*_2,52_ = 7.62, p=0.001) but no main effect of Treatment (*F*_1,26_ = 2.6, p>0.05). Post hoc analysis revealed that rats injected with R12 pressed significantly less for cues in the final bin of the Day 30 session compared to vehicle injected rats (*t*_26_ = -4.62, p < 0.001).

*Experiment 2*

Similar to *Experiment 1*, we compared the terminal responding (self-administration Sessions 3,4,5 for pre-dependence and Sessions 13,14,15 for post-dependence) for infusions + cues earned and lever pressing between the two cohorts. A repeated measures ANOVA with Sessions (3-5: pre-dependence) as the within subject factor and cohort as between subject factor showed a significant effect of Session (*F*_2,88_= 4.36, p=0.02), and cohort (*F*_1,44_= 5.17, p=0.028) but no interactions (*F*_2,88_= 0.04, p > 0.05) for infusions + cues. Notably, the cohort difference in infusions + cues earned during pre-dependence phase was no longer evident post-dependence. For post-dependence Sessions 13-15, we did not observe any main effects or interactions (*F*’s < 0.9, p’s > 0.05) for infusions + cues earned. Next, a repeated measure ANOVA with Lever (Active, Inactive) and Sessions (3-5: pre-dependence) as within subject factors and cohort (1,2) as between subject factors showed a main effect of Lever (*F*_1,44_= 17.29, p<0.001), Session (*F*_2,88_= 8.48, p=0.001), a significant Lever x Session interaction (*F*_2,88_= 8.03, p<0.001) but no main effect of cohort or other interactions (*F*’s <2.1, p’s > 0.05). Further, when we compared terminal lever pressingbetween the cohort’spost-dependence, there was only a main effect of lever (*F*_1,44_= 39.53, p<0.001) but no other main effects or interactions (*F*’s <1.18, p’s > 0.05). Hence, we pooled the two cohort’s data together.

*Experiment 2: Protracted withdrawal (Day 5 vs Day 30 test)*:

Supplementary Table 1 showscues earned effect sizes (day 5 (vehicle, vehicle) vs. day 30 (vehicle, R121919)) for dependenceand Day 30 treatment groups. Only rats that were injected with R121919 on Day 30 test showed the large effect sizes (bold text).

**Supplementary Table 1**

*Effect sizes for cues earned in acute (Day5) vs protracted (Day30) withdrawal*

| Dependence |  | Dependent | | Non-dependent | | |  | Both |  |
| --- | --- | --- | --- | --- | --- | --- | --- | --- | --- |
|  | *Mean* | *SD* | *Cohen’s d* | *Mean* | *SD* | *Cohen’s d* | *Mean* | *SD* | *Cohen’s d* |
| Day 5 treatment  Vehicle  Vehicle | 36.5  44.4 | 25  17 |  | 31.75  29.25 | 11  12 |  | 34.12  36.88 | 18.3  16.1 |  |
| Day 30 treatment |  |  |  |  |  |  |  |  |  |
| Vehicle | 27.5 | 12 | 0.45 | 31.75 | 19 | 0 | 29.63 | 15 | 0.29 |
| R121919 | 22 | 12 | **1.52** | 16.25 | 7.1 | **1.28** | 19.13 | 9.6 | **1.34** |

The lever press data (Supplementary Fig. 2B,C), followed a similar pattern to cues earned, such that all rats responded less on the active lever during Day 30 compared to Day 5 tests (*F*_1,12_= 20, p=0.001). The Lever main effect (*F*_1,12_= 41.57, p<0.001) and a Withdrawal Day x Lever interaction (*F*_1,12_= 18, p=0.001) indicated discrimination of active and inactive levers, with a specific reduction in active responding between withdrawal test days, but no main effects or interactions for Dependence or Day 30 Treatment (*F*’s < 1.11, p’s > 0.05).

*Acute Withdrawal (Day 5 test)*:

Rats injected with intra-BNST R121919 showed a trend in attenuation of lever presses compared to vehicle injected rats (Supplementary Fig. 3C). A mixed ANOVA resulted in a main effect of Lever (*F*_1,24_= 26.11, p<0.001) and amain effect of Day 5 Treatment (*F*_1,24_= 4.52, p=0.044)but no other main effects or interactions (*F*’s <2.31, p’s > 0.05). In addition, intra-BNST R121919 treated rats tended to press the active lever less vigorously (Supplementary Fig. 3D). An ANOVA on active lever press rate resulted in a trend inmain effect of Day 5 Treatment (*F*_1,24_= 3.38, p=0.079) but no main effect of Dependence (*F*_1,24_= 1.35, p>0.05) or a Day 5 Treatment x Dependence interaction (*F*_1,24_= 0.09, p>0.05). An ANOVA on time out responses (*Active Lever presses – cues earned*) also trended towards significancefor Day 5 Treatment (*F*_1,24_= 2.85, p=0.10) but no main effect of Dependence (*F*_1,24_= 1.30, p>0.05) or a Day 5 Treatment x Dependence interaction (*F*_1,24_= 0.05, p>0.05) (Supplementary Fig. 3E). These results suggest that both dependent and non-dependent rats show a trend in fewer lever presses, lower press rate, and fewer time out presses when BNST CRFR1 are antagonized.

*Opioid demand:*We ran a paired samples *t*test between intra-BNST vehicle and intra-BNST R121919sessionsforfour different measures: remifentanil consumption at low cost (Q_0_), demand elasticity (α), total remifentanil consumption, and total active lever presses(Supplementary Fig. 4A-D). We did not see a significant difference in any of these measures, Q_0_: *t*_8_ = 0.71, p > 0.05, α: *t*_8_ = 0.32, p > 0.05, total remifentanil consumption: *t*_9_ = 0.77, p > 0.05, and total Active Lever presses: *t*_9_ = 0.88, p > 0.05.

We compared Active Lever presses in the last bin of behavioral demandsessions (when the dose of remifentanil administered is extremely low and non-reinforcing) between vehicle and R121919 injected conditions (Supplementary Fig. 4E). We observed no difference (paired *t* test between intra-BNST vehicle and intra-BNST R121919: *t*_10_  = 1.69, p > 0.05) in the last bin Active Lever presses, suggesting that immediately after periods of drug reinforcement when rats are reinforced by cues alone, intra-BNST infusions of R121919 have no effect.

**Supplementary Figure captions**

Figure S1

**A)**Time course of lever presses on Day 1 incubation test, in which all rats received intra-BNST vehicle injections,plotted by rats’prospective Day 30 vehicle (black) andDay 30 R121919 groups (gray).Veh = Vehicle, R12 = R121919. Data are mean±SEM.**B)** Time course of cues earned on Day 30 incubation test between vehicle and R121919 treatedgroups. R12 injected rats pressed significantly less for cues in the final bin of the incubation test. *p<0.05.

Figure S2

Incubation of fentanyl craving in opioid dependent and non-dependent rats after protracted withdrawal (Day5 vs Day30 test). **A-C)**Incubation test data showing cues earned on FR1, 20 s TO schedule.**(A)**Active Lever presses.**(B)** and Inactive Lever presses.**(C)**Veh = Vehicle, R12 = R121919, Dep = Dependent, Non-dep = Non-dependent. Data are mean±SEM.

Figure S3

**(A)** Bout size (number of presses per bout). **(B)** Bout duration (time from first to last press in a bout). **(C)** Inter bout interval, the time between last press in bout and first press in next bout. **(D)** Day 5 Incubation test data showing total lever presses (Active and Inactive). **(E)** Active lever press rate. **(F)** Time out responding (*Active Lever presses-cues earned*). All the inset graphs indicate mean ± SEM when collapsed across dependence. Veh = Vehicle, R12 = R121919, ND = Non-dependent. D = Dependent. Data are mean ± SEM.

Figure S4

**A)**Log_10_ Q_0_, remifentanil consumption at low price. **B)** Alpha (α), demand elasticity **C)**Total remifentanil consumed throughout the 150 min session. **D)**Total Active Lever presses. **E)** Active Lever presses in the last bin of demandsessions. ns = not significant.Veh = Vehicle, R12 = R121919. Data are mean±SEM.
